# Evaluation of the Effects of R-CHOP Chemotherapy on Brain Glucose Metabolism in Patients with Diffuse Large B Cell Lymphoma: A Baseline, Interim, and End-of-Treatment PET/CT Study

**DOI:** 10.3390/tomography8050214

**Published:** 2022-10-12

**Authors:** Haiyan Zhu, Fei Li, Yan Chang, Yabing Sun, Nan Wang, Ruimin Wang

**Affiliations:** 1 Department of Hematology, The Fifth Medical Center, Beijing 100039, China; 2 Department of Nuclear Medicine, The First Medical Center, Chinese PLA General Hospital, Beijing 100853, China

**Keywords:** brain glucose metabolism, PET/CT, R-CHOP, DLBCL

## Abstract

Background: To investigate the effect of rituximab, cyclophosphamide, doxorubicin, vincristine, and prednisone (R-CHOP) chemotherapy on brain glucose metabolism in patients with diffuse large B cell lymphoma (DLBCL). Methods: Seventy-two patients with newly diagnosed DLBCL underwent FDG PET/CT brain and whole-body scans at baseline (PET0), in the interim of chemotherapy (PET2), and at the end (PET6) of chemotherapy. All three brain scans of each patient were analyzed using statistical parametric mapping software. Results: Compared with the PET0 scan, the PET2 and PET6 scans revealed a significantly higher glucose metabolism throughout the whole brain, with the PET6 scan revealing a higher metabolism than the PET2 scan. Patients with a complete response (CR) displayed decreased glucose metabolism in the lingual gyrus and increased glucose metabolism in the pons after chemotherapy compared with the findings in patients with partial responses or progressive disease. Conclusions: Brain glucose metabolism was affected by R-CHOP treatment throughout the entire chemotherapy protocol.

## 1. Background

Diffuse large B cell lymphoma (DLBCL) is the most common and aggressive subtype of non-Hodgkin Lymphoma (NHL), and positron emission tomography/computed tomography (PET/CT) using fluorine ^18^F-fluoro-2-deoxy-d-glucose (^18^F-FDG) labeling is a widely accepted method for assessing responses to first-line treatment including rituximab, cyclophosphamide, doxorubicin, vincristine, and prednisone (R-CHOP) in patients with DLBCL [1]. More attention is paid to the metabolic changes in lymphoma lesions in the body during treatment, whereas the effect of chemotherapy on brain metabolism and cognitive complications are generally neglected. The patients who receive chemotherapy sometimes complain of memory loss, depression, anxiety, and other symptoms of cognitive dysfunction. Several studies demonstrated that neurotoxicity related to chemotherapy regimens plays a role in these symptoms [2]. Currently, the evaluation of the effect mainly relies on neurophysiological scales and other grading tools [3].

However, only a few studies used imaging modalities such as magnetic resonance imaging to assess changes in the brains of solid tumor survivors [4] or patients with lymphoma [5]. Conversely, the mechanism of this impairment, the associated metabolic changes, and the relationship between changes in the brain and the patient’s prognosis have not been clarified, and the results of the reported studies have also been inconsistent [6,7]. Because the head is often included in the scan range of PET/CT in patients with lymphoma in our center not only due to the conventional scan protocol including the whole body and brain, but also to exclude the tumor invasion of brain tissue, this provides an opportunity to study brain glucose metabolism during the treatment process.

Therefore, we retrospectively investigated the use of FDG PET/CT to measure brain glucose metabolism before chemotherapy and after two and six cycles of chemotherapy in patients with newly diagnosed DLBCL. The purpose of the research was to explore the changes in brain glucose metabolism visually throughout the entire chemotherapy period and whether these changes after chemotherapy could predict treatment efficacy.

## 2. Methods

### 2.1. Patients

This retrospective study was approved by the Ethics Committee of the local hospital. All subjects provided written informed consent. From January 2009 to September 2014, 72 patients with newly diagnosed DLBCL were enrolled in this study. The patient’ inclusion criteria were as follows: de novo pathologically proven DLBCL; receipt of first-line chemotherapy with rituximab, cyclophosphamide, doxorubicin, vincristine, and prednisone (R-CHOP); FDG PET/CT scans performed before (PET0) and after two (PET2) and six cycles (PET6) of chemotherapy; and the availability of images in a digital format for review and analysis. The exclusion criteria were as follows: progression of transformed lymphoma-like follicular into DLBCL and/or another malignant disease; presence of lymphoma lesions in the central nervous system; history of neuropsychiatric disease; and receipt of any salvage therapy other than R-CHOP.

### 2.2. PET/CT Scans

A GE Discovery VCT PET/CT scanner (GE Healthcare, Milwaukee, WI, USA) was used to assess the distribution of ^18^F-FDG in the brain and the whole body of the subjects. All patients received intravenous ^18^F-FDG (4–5 MBq/kg) after fasting for at least 6 h. Patients were required to remain in a resting condition for 60 min, and then PET scanning was performed from the neck to the upper thigh (whole body scan). A brain scan was separately taken after the whole-body scan by placing the patient’s head in a dedicated support, and a 5 min emission scan was performed after the CT scan for attenuation correction (120 kV, 110 mA). The PET data were reconstructed using the VUE Point HD system (GE Medical Systems) in the 3D mode in a 192 × 192 matrix.

The time point for the PET2 scan was as close as possible to the beginning of the third cycle of chemotherapy, and the PET6 scan was conducted at least 1 month after the end of chemotherapy.

### 2.3. PET/CT Data Analysis

FDG PET/CT brain image data were analyzed using statistical parametric mapping (SPM) software (SPM8; Wellcome Trust Centre for Neuroimaging, Institute of Neurology, University College London, London, UK) operated within MATLAB 7.4.0.287 (MathWorks, Natick, MA, USA) for Windows. For this purpose, images in the DICOM format were converted into an analyzable format using MRIcro 1.40 (http://www.nitrc.org/projects/mricro/) (accessed on 21 February 2021)). Spatially normalized images were mapped onto a PET-specific template and then smoothed via convolution using a Gaussian kernel with an 8 mm full-width half-maximum. A paired *t*-test was used to compare brain images between groups (PET0 vs. PET2, PET0 vs. PET6, PET2 vs. PET6). SPM coordinates were corrected to match the Talairach coordinates. Clusters containing more than 125 voxels (5 × 5 × 5 voxels, cluster size: 11 mm × 11 mm × 11 mm) were accepted as significant because a smaller threshold can ensure that as many small differences as possible are recognized, and *p* values were corrected for multiple comparisons using family-wise error rates. Corrected *p* < 0.05 denoted statistical significance. When statistically significant differences were not identified at this conservative threshold, uncorrected *p* < 0.01 or *p* < 0.001 served as the significance threshold.

## 3. Results

### 3.1. Patients and Characteristics

The patient characteristics are listed in Table 1. According to the Lugano classification, the follow-up data at the end of six cycles of chemotherapy revealed complete responses (CR), progressive disease (PD), and partial responses (PR) in 55, 7, and 10 patients, respectively.

### 3.2. Comparison of Brain Metabolism at Baseline (PET0) and in the Interim of (PET2) Chemotherapy

**Figure 1:**
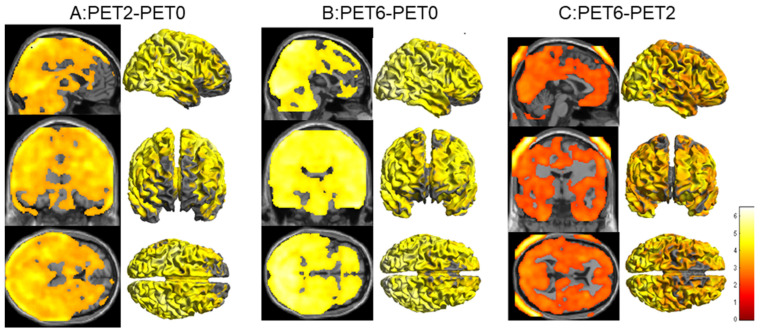
(**A**): Statistical parametric mapping (SPM) analysis revealed significantly increased brain metabolism in nearly the whole brain, excluding the bilateral prefrontal cortices and ventricle (overlaid in the MRI T1-weighted image and 3D cortex template), in the PET2 scan compared with that in the PET0 scan (*p* < 0.001, uncorrected). (**B**): SPM analysis revealed increased brain metabolism in the PET6 scan compared with that in the PET0 scan (*p* < 0.001, uncorrected). (**C**): SPM analysis revealed further increases in brain metabolism in the PET6 scan compared with that in the PET2 scan (*p* < 0.05, uncorrected).

There was a significant increase in metabolism involving nearly the whole brain at PET2 compared to PET0, excluding the bilateral prefrontal cortices and ventricles, and there were no areas of the brain where a significant reduction in FDG metabolism occurred (Figure 1A).

### 3.3. Comparison of Brain Metabolism at Baseline (PET0), in the Interim of (PET2), and at the End of (PET6) Chemotherapy

There was a significant increase in metabolism involving nearly the whole brain at PET6 compared to PET0, excluding a small portion of the prefrontal cortices and ventricles (Figure 1B). There were also no areas of the brain where a significant reduction in FDG metabolism occurred.

Furthermore, brain glucose metabolism was higher in the PET6 scan than in the PET2 scan for the whole brain, excluding the bilateral white matter around the ventricles, brainstem, and cerebellum (Figure 1C).

### 3.4. Correlations between Brain Glucose Metabolism Changes and Therapeutic Responses

**Figure 2:**
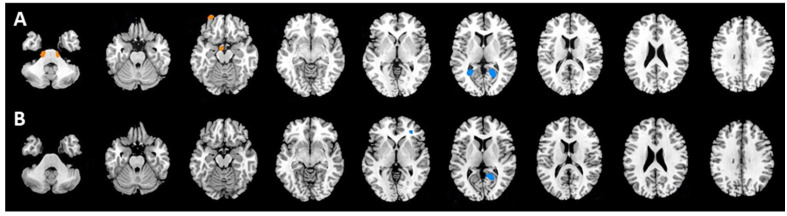
(**A**): Patients with complete responses (CRs) exhibited hypermetabolism on both sides of the pons (red) and hypometabolism on both sides of the lingual gyrus (blue) compared with the findings in the NonCR group (partial responses and progressive disease) in the PET2 scan. (**B**): The CR group displayed only hypometabolism in the left lingual gyrus and inferior frontal gyrus compared with the findings in the NonCR group in the PET6 scan.

In the PET2 scan, the group of 55 patients with CRs at the end of chemotherapy exhibited hypermetabolism on both sides of the pons and hypometabolism on both sides of lingual gyrus compared with the findings in the 17 patients with PRs or PDs (NonCR group). In the PET6 scan, the CR group displayed hypometabolism only in the left lingual gyrus and inferior frontal gyrus compared with the findings in the NonCR group. Figure 2 and Table 2 presented the areas and corresponding Talairach coordinates with significant differences in brain glucose metabolism in the CR and NonCR groups in the PET2 (Figure 2A) and PET6 (Figure 2B) scans.

## 4. Discussion

The patients who have received chemotherapy may suffer from impaired cognition named chemobrain. The cognitive impairment includes changes in verbal and visual memory, attention, concentration, language, and motor skills. The mechanisms of the neurotoxicity are not well understood. Several explanations have been postulated including increased expression of tumor necrosis factor alpha induced by chemotherapy [8], which, together with oxidative stress and apoptosis, inhibits neuronal proliferation and differentiation [9]. Most studies have mainly been conducted in patients with cancers such as breast, rectal, and lung cancers. A few previous studies focused on lymphoma, especially DLBCL and Hodgkin’s lymphoma (HL) by means of evaluating clinical symptoms and psychological tests [10]. FDG PET/CT can be used to evaluate physiological processes in the body including glucose consumption in the brain, which provides objective visual evidence of the effects of chemotherapy. Because the brain is often included in the range of the PET/CT scan in patients with DLBCL, this permits the study of changes in glucose metabolism in the brain during the treatment period. To our knowledge, this study represents the largest analysis of DLBCL patients who underwent PET/CT both during and after chemotherapy and assessments of brain glucose metabolism via SPM analysis.

The results of previously reported studies of brain metabolism changes in patients with lymphoma have been contradictory. Chiaravalloti et al. [11]. reported significantly higher metabolic activity in the right angular gyrus and significantly lower metabolic activity in the bilateral prefrontal and orbitofrontal cortices and left anterior cingulate cortex after two cycles of Adriamycin, bleomycin, vinblastine, and dacarbazine (ABVD) chemotherapy in patients with Hodgkin’s lymphoma (HL). Nonokuma et al. [12]. demonstrated significantly increased metabolic activity in the bilateral parietal and occipital regions and decreased metabolic activity in the bilateral cerebellar hemisphere, right putamen, bilateral insula, and bilateral anterior cingulate regions after treatment in 30 patients, including 16 patients with DLBCL. Conversely, a study by Adams et al. [13]. concluded that brain glucose metabolism was not affected by R-CHOP therapy in patients with DLBCL.

We could not clarify the mechanisms of chemobrain in patients with DLBCL using FDG PET/CT scans, but the changes in brain glucose metabolism to some extent reflect the effects of R-CHOP therapy. The explanations of the decreases in cerebral glucose metabolism reported in some studies after chemotherapy are uncertain. Several possible reasons for decreases in metabolic activity in chemotherapy-treated patients have been postulated as follows: chemotherapeutic drug penetration of the blood–brain barrier (BBB) during chemotherapy [14]; an autoimmune reaction, named paraneoplastic syndrome, caused by cytokines secreted from activated immune system cells [15]; the intravascular invasion of lymphoma cells and occlusion of the arterioles, capillaries, and venules caused by cell proliferation [16]; post-traumatic stress disorder (PTSD) caused by a cancer diagnosis and depressive symptoms caused by the stress of having a life-threatening disease [17]; and other uncertain explanations.

Although most studies reported decreased brain metabolism after chemotherapy, a few observed higher metabolic activity in the angular gyrus after the second cycle of chemotherapy in patients with HL [11], and increased FDG uptake was noted after treatment in the parietal and cingulate cortices of patients with NHL [18]. The most distinctive result of our study was the significant hypermetabolism found in nearly the whole brain, excluding the bilateral prefrontal cortices and ventricles during chemotherapy, and further increases in metabolic activity were observed at the end of treatment. Because of the limited reported evidence and experience, the possible reasons for this difference, based on our knowledge, are as follows: as cerebral glucose metabolism is affected by the mental state of the patient, PTSD can play a role even after two and six cycles of treatment, and increased attention or fear responses in patients lead to metabolic alterations in regions of the brain cortex such as the parietal lobe. Patients are still shocked and uncertain by the diagnosis at the time of the PET0 scan, and they are anxious regarding the therapeutic outcome during the PET2 scan and worried about the possibility of disease recurrence during the PET6 scan. Moreover, pre-treatment scans always exhibit a reduced cerebral glucose metabolism, and most patients with DLBCL (55% in our study) exhibit CRs at the end of treatment. This therefore suggests that regional metabolic changes in the cerebrum are caused by the redistribution of FDG. In other words, FDG shifts from lymphoma lesions to the brain because of the reduced tumor burden in the body. On the contrary, most reports identified a decreased (and/or increased) metabolism in cerebral regions after ABVD chemotherapy in patients with HL, a non-specific subtype of NHL, and there was no consensus concerning cerebral metabolic changes associated with the R-CHOP regimen in patients with DLBCL. Different drugs, based on their ability to penetrate the BBB, may lead to specific chemotherapy-related cytotoxicity. This indicated that R-CHOP, a regimen including one neurotoxic agent (vincristine) and one BBB-penetrating drug (prednisone), affected the subjective perception of cognition after chemotherapy [19]. In addition, the increased levels of pro-inflammatory cytokines (e.g., IL-1Ra, CRP, IL-6) during chemotherapy were found to correlate with greater brain glucose metabolism [20]. In addition to the aforementioned explanations, the different results between our study and previous research could be attributable to heterogeneity concerning experimental methodologies. Because no specific brain region is known to be protected against the effects of chemotherapy, the FDG update in the entire brain was analyzed opposed to that in specific regions. Thus, the SPM methodology, to some extent, is more objective than visual inspection and standardized the uptake value calculation performed using regions of interest, in which diffuse changes in brain metabolism might be underestimated [18].

Moreover, the therapeutic response and prognostic value of brain glucose metabolism measurements are currently unclear for patients with DLBCL. Our results provide evidence that some focal brain regions exhibit hyper/hypometabolism, which may reflect the response to R-CHOP treatment during and after chemotherapy. However, only a few published studies have indicated a possible relationship between a low pre-treatment brain glucose metabolism and worse outcomes [13]. The reason for this is that prior research only concentrated on the pre-treatment status regarding survival opposed to the post-treatment status concerning the therapeutic response. The significant changes in metabolism in some brain regions, namely, the pons and lingual gyrus, could reflect the therapeutic response based on our results. Specifically, a decreased glucose metabolism in the lingual gyrus and an increased glucose metabolism in the pons after chemotherapy is possibly related to good treatment responses. The lingual gyrus and pons are involved in logical analysis, visual memory, and sleep regulation. We have no such experience with nor could we find published data to explain this manifestation in patients with DLBCL. However, a similar finding that the lingual gyrus might be a possible candidate region for predicting the response to antidepressants and the maintenance of cognition was reported in patients with major depressive disorder [21]. Furthermore, our results may provide objective evidence for future research on the relationship between cerebral metabolism and prognosis in patients with cancer.

The present study had several limitations. First, as this was a retrospectively study, the patients’ mental state and performance were not evaluated objectively. We only could find the patients’ complaints or symptoms from the medical records, whereas the hematologist failed to consistently notice mild-to-moderate neuropsychological symptoms during treatment and record such findings in a timely manner. Hence, the main limitation is the lack of clinical neuropsychological data such as questionnaire data, even though 17–75% of all patients with cancer experience psychological side effects from cancer therapies [22]. Second, the changes in brain metabolism involve a complex web of interactions including the patient’s internal status and external influences, in addition to chemotherapy. It is difficult to precisely assess any specific phenomenon using functional imaging methods. Third, the results of SPM analysis, derived from computer software, represent relative values of brain glucose metabolism, differing from absolute quantitative assessments.

## 5. Conclusions

Brain glucose metabolism as assessed via FDG PET/CT continued to increase in most brain regions during R-CHOP chemotherapy. The decreased glucose metabolism in lingual gyrus and increased glucose metabolism in the pons after chemotherapy are possibly related to poor treatment responses. Further studies integrating imaging modalities with clinical neuropsychological evaluation and laboratory assessments are necessary to confirm the existence of neurotoxicity, and these studies may support the development of appropriate treatment strategies for patients with cancer.

## Figures and Tables

**Table 1:** Characteristics of the 72 patients with diffuse large B cell lymphoma.

Characteristic	Data
Age (mean ± standard deviations)	47.8 ± 16.7 (18–81)
No. of male patients	40 (55.6%)
No. of female patients	32 (44.4%)
Ann Arbor Stage	
I–II	35 (48.6%)
III–IV	37 (51.4%)
IPI	
Low risk (0–1)	44 (61.1%)
Low-intermediate risk (2)	14 (19.4%)
High-intermediate risk (3)	10 (13.9%)
High risk (4–5)	4 (5.6%)
B symptoms	
Positive	31 (43.1%)
Negative	41 (56.9%)
Germinal center (GC)	
GC	30 (41.7%)
Non-GC	42 (58.3%)

Abbreviation: IPI, international prognostic index.

**Table 2:** Brain areas with significantly different glucose metabolism between the complete response (CR) and NonCR (progressive disease and partial response) groups in the PET2 and PET6 scans.

Comparison	Region	Talairach Coordinates			T Score	*p* Values (Uncorrected)
		x	y	z		
CR&NonCR (PET2)						
hypermetabolism	Right pons 1	10	−26	−50	1.96	0.026
	Right pons 2	16	−24	−44	1.95	0.027
	Left pons	−14	−24	−40	1.76	0.041
hypometabolism	Right lingual gyrus	28	−56	8	2.12	0.018
	Left lingual gyrus	−20	−56	8	2.33	0.011
CR&NonCR (PET6)						
hypometabolism	Left lingual gyrus	−18	−54	8	2.30	0.011
	Left inferior frontal gyrus	−32	−38	2	2.55	0.006

## Data Availability

The datasets used and analyzed in the current study are available from the corresponding author on reasonable request.

## Abbreviations

DLBCL: Diffuse large B cell lymphoma; NHL: Non-Hodgkin Lymphoma; HL: Hodgkin Lymphoma; R-CHOP: Rituximab, cyclophosphamide, doxorubicin, vincristine, and prednisone; PET/CT: Positron emission tomography/computed tomography; ^18^F-FDG: ^18^F-fluoro-2-deoxy-d-glucose; PET0(2, 6): PET/CT scans performed before (after two or six cycles of) chemotherapy; SPM: Statistical parametric mapping; CR: Complete response; PD: Progressive disease; PR: Partial response; NonCR: PD and PR.
